# Early Decrease in Respiration and Uncoupling Event Independent of Cytochrome *c* Release in PC12 Cells Undergoing Apoptosis

**DOI:** 10.1155/2012/643929

**Published:** 2012-05-16

**Authors:** Libera Berghella, Elisabetta Ferraro

**Affiliations:** ^1^Pathophysiology and Treatment of Cachexia Unit, IRCCS San Raffaele Pisana Institute, 00166 Rome, Italy; ^2^Division of Biology, California Institute of Technology, Pasadena, CA 91125, USA

## Abstract

Cytochrome *c* is a key molecule in mitochondria-mediated apoptosis. It also plays a pivotal role in cell respiration. The switch between these two functions occurs at the moment of its release from mitochondria. This process is therefore extremely relevant for the fate of the cell. Since cytochrome *c* mediates respiration, we studied the changes in respiratory chain activity during the early stages of apoptosis in order to contribute to unravel the mechanisms of cytochrome *c* release. We found that, during staurosporine (STS)- induced apoptosis in PC12 cells, respiration is affected before the release of cytochrome *c*, as shown by a decrease in the endogenous uncoupled respiration and an uncoupling event, both occurring independently of cytochrome *c* release. The decline in the uncoupled respiration occurs also upon Bcl-2 overexpression (which inhibits cytochrome *c* release), while the uncoupling event is inhibited by Bcl-2. We also observed that the first stage of nuclear condensation during STS-induced apoptosis does not depend on the release of cytochrome *c* into the cytosol and is a reversibile event. These findings may contribute to understand the mechanisms affecting mitochondria during the early stages of apoptosis and priming them for the release of apoptogenic factors.

## 1. Introduction

Mitochondria play a key role in apoptosis triggered by a wide variety of stimuli since they release important proapoptotic factors from their intermembrane space. The first mitochondrial apoptogenic molecule discovered was the hemoprotein cytochrome *c* [1]. Cytochrome *c* is involved in two critical cell processes. Normally, it acts as mobile electron carrier shuttling electrons between ubiquinol cytochrome *c* oxidoreductase (complex III) and cytochrome *c* oxidase (complex IV) of the respiratory chain allowing cell life. On the other hand, upon apoptotic induction, cytochrome *c* is released from mitochondria in the cytosol where it carries out a proapoptotic function by binding the adapter protein apoptosis protease-activating factor-1 (Apaf1). Consequently, it promotes, in presence of ATP/dATP, the assembly of the multiproteic complex apoptosome, which binds and activates the caspase-9, thereby initiating the activation of a caspase cascade which leads to apoptotic cell death [2, 3].

Mitochondrial outer membrane permeabilization (MOMP) and cytochrome *c* release from mitochondria during apoptosis are tightly regulated by the proteins of the Bcl-2 family. This family of proteins includes both antiapoptotic members (e.g., Bcl-2 and Bcl-XL) repressing MOMP and release of apoptogenic factors from mitochondria and pro-apoptotic members promoting MOMP (e.g., Bax and Bak, Bid) [4, 5]. However, the detailed mechanisms of cytochrome *c* release are still unclear.

The phospholipid cardiolipin (CL) seems to have a key role in the process of the cytochrome *c* release [6]. Only 15% of cytochrome *c* is free in the intermembrane space, while most of it is attached to the outer leaflet of the mitochondrial inner membrane with the mitochondrion-specific anionic phospholipid CL. It has been recently found that posttranslational modifications or interaction with hydrophobic anions such as CL causes the activation of cytochrome *c* into a peroxidase with selective catalytic competence toward CL [7, 8]. Cytochrome *c* tightly bound to CL was proposed to possess this peroxidase activity and to catalyze CL peroxidation, most likely by utilizing the high production of reactive oxygen species (ROS) generated in the mitochondria during the first stages of apoptosis. Cytochrome *c* has a lower affinity for peroxidized CL, and peroxidation of CL enables the dissociation of cytochrome *c* from mitochondrial inner membrane allowing the release of cytochrome *c* from mitochondria. CL oxidation is, indeed, mandatory for the release of cytochrome *c* [6]. However, the final release of cytochrome *c* requires additional steps such as the permeabilization of the outer membrane. CL is also involved in mitochondrial outer membrane permeabilization since it enables docking and activation of some pro-apoptotic Bcl-2 proteins [9–11].

These findings strongly suggest an active role of the membrane in modulating MOMP that has been underestimated so far. Cytochrome *c* is part of the respiratory chain whose complexes are located across the mitochondrial inner membrane, and membrane integrity greatly influences the respiratory activity of the cells. Based on this considerations, we analyzed the respiratory changes during apoptosis and their temporal relationship with the release of cytochrome *c* in order to get information useful to unravel the mechanisms of release of this hemoprotein from mitochondria. We therefore analyzed the respiratory activity of a rat cell line of neuronal derivation (pheochromocytoma-12, PC12 cells) by polarographic measurement of oxygen consumption in intact cells [12]. The PC12 line was originally cloned from a transplantable rat adrenal medullary pheochromocytoma. This cell line manifests many features of sympathicoblasts, the cells that give rise to postmitotic sympathetic neurons. Indeed, they respond to nerve growth factor (NGF) by shifting to a nonproliferating neurite-bearing phenotype and acquiring many of the properties characteristic of sympathetic neurons among which electrical excitability. For this reason the clonal PC12 cell line is widely used for various studies on neuronal cell differentiation and function. During staurosporine- (STS-) induced apoptosis in naïve PC12 cells, we observed an uncoupling event preceding the reduction of cytochrome *c* oxidase- (COX-) respiratory activity. Our investigation has also revealed different kinetics of decrease in 2,4-dinitrophenol- (DNP-) uncoupled and COX-dependent respiration with the former showing, at very early stage, a faster kinetics of decrease compared with the latter. This suggests an effect of STS on the respiratory activity, which is independent of cytochrome *c* release. This hypothesis is confirmed by our finding that overexpression of Bcl-2 protects from release of cytochrome *c* and massive decrease in respiration, while it has no effect on the early decrease in DNP-uncoupled respiration induced by STS.

## 2. Results

### 2.1. Measurement of Respiration in Intact STS-Treated PC12 Cells Early Decrease in DNP-Uncoupled Respiration

Since the cytochrome *c* is part of the respiratory chain as the electron donor for complex IV, it is expected that its release from mitochondria during apoptosis would cause changes in the respiratory activity of the cells. We analyzed the nature and the extent of these changes by measuring the rate of oxygen consumption in naive PC12 cells and in naive PC12 cells undergoing apoptosis by treatment with the protein kinase inhibitor STS [13, 14].

**Figure 1:**
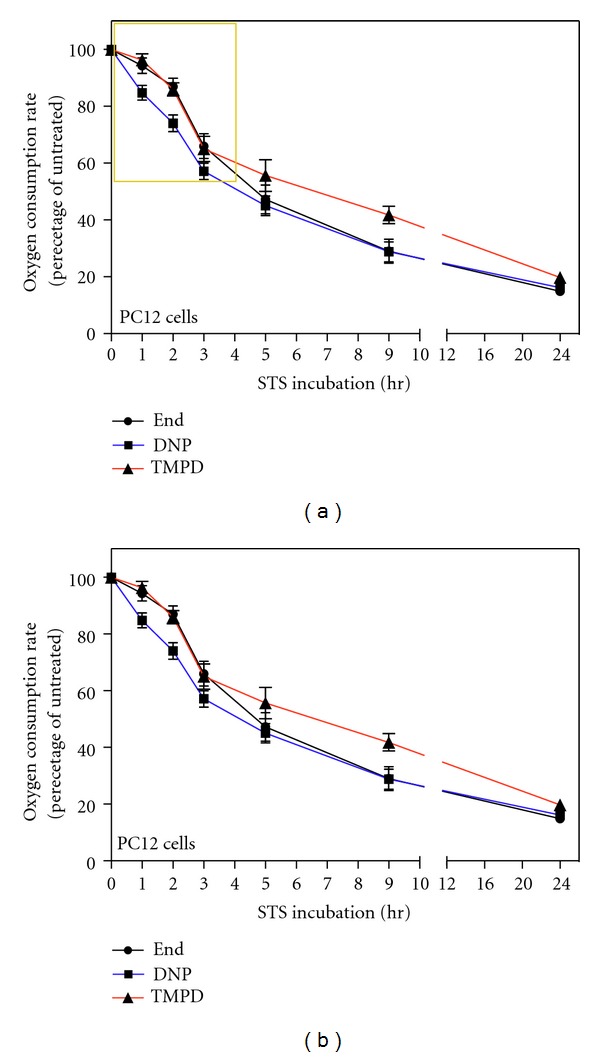
Decrease in endogenous, DNP-uncoupled, and TMPD-dependent respiration rates in intact PC12 cells treated with STS; early decrease in DNP-uncoupled respiration. (a) Oxygen consumption rates of 2 *μ*M STS-treated PC12 cells measured at the indicated time points. Oxygen consumption rates were measured in TD buffer (endogenous respiration, End), in TD buffer containing DNP (DNP-uncoupled respiration, DNP) and in TD buffer containing DNP, antimycin A, ascorbate, and TMPD (TMPD-dependent respiration, TMPD). Data are expressed, at each time point, as percentages of the oxygen consumption of untreated cells (0 hr). To reduce the variability of the experiments we calculated the percentages with respect to the control of that particular experiment, and then we averaged the percentages. Data are expressed as means ± SE (standard error) with *n* varying at each time point and being 31, 16, 9, 6, 7, 2, and 7, respectively, for the indicated time points (1 h, 2 h, 3 h, 5 h, 9 h, 24 h). (b) Magnification of (a) area surrounded by a yellow frame. Note that the ordinate scale starts at 50%.

**Figure 2:**
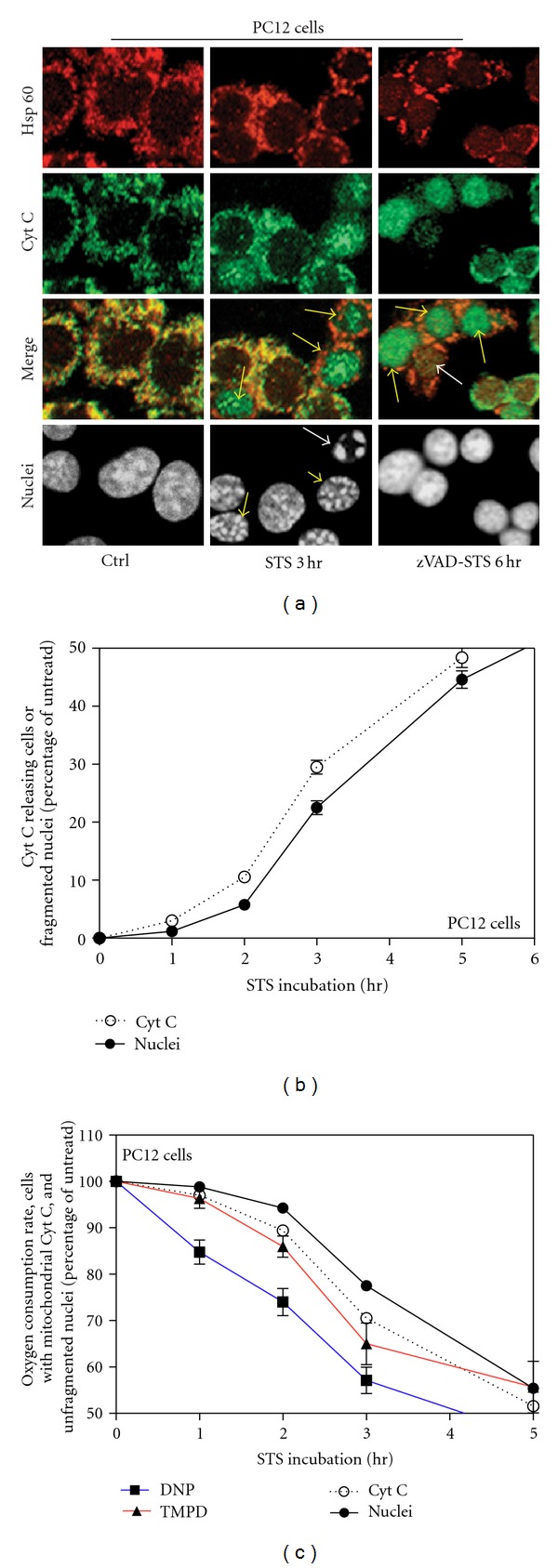
Cytochrome *c* release and nuclear apoptosis in STS-treated PC12 cells. (a) Triple labeling confocal immunofluorescence microscopy of cells untreated (Ctrl), 2 *μ*M STS-treated for 3 h (STS 3 h), or 2 *μ*M STS-treated for 6 h with 30 min zVADfmk (100 uM) pretreatment (zVAD-STS 6 h). Pattern of Hsp60 (red), cytochrome *c* (green), merged patterns (yellow) and nuclear staining by SYTO-X (grey) of the same representative fields are shown. (b) Percentage of cytochrome *c* releasing and apoptotic PC12 cells treated with STS for the indicated time. Data calculated in 3 independent experiments for each time point. About 1000 cells were considered for each experiment at each time point. Some SE are small and are within the symbols. Note that the ordinate scale starts at 50%. (c) Comparison between data shown in Figure 2(b) but expressed as decrease of cells with mitochondrially localized cytochrome *c* and decrease of alive cells and data regarding decrease in respiration shown in Figure 1(b).

 We measured the oxygen consumption of the cells (endogenous respiration), the oxygen consumption in presence of the uncoupler DNP (DNP-uncoupled respiration, which represents the highest endogenous respiration rate that cells can reach being free of control by the proton gradient) and the rate of respiration measured in presence of the complex III inhibitor antimycin A, the artificial membrane-permeant electron donor TMPD (N,N,N′,N′-tetramthyl-1,4-phenylendiamine), and the reducing agent ascorbate that maintains TMPD in a reduced state (TMPD-dependent respiration) [12]. Since antimycin A inhibits complex III and blocks the electron flux upstream of COX, the TMPD-dependent respiration provides a measure of the COX and cytochrome *c*-dependent oxygen consumption independently of the upstream segment of the respiratory chain [15]. The respiration of both untreated and STS-treated PC12 cells resulted being 100% antimycin A-sensitive (data not shown). Upon treatment with STS we observed a time-dependent decrease in the endogenous, DNP-uncoupled and TMPD-dependent respiration rates. After 5 hr of treatment with STS, the DNP-uncoupled respiration rate decreased to ~45% of the DNP-uncoupled respiration rate of untreated cell and to ~28,8% after 9 hr of treatment (Figures 1(a) and 2(b)). The oxygen consumption relative to prolonged period of STS treatment (9 h and 24 h) is overestimated since, being very light, it is difficult to collect all dying cells with the usual centrifugation speed; and it is not advisable to use higher speed since it would affect the respiration of alive cells.

The kinetics of decrease in TMPD-dependent COX respiration rate during STS-treatment was similar to the kinetics of decrease in endogenous and DNP-uncoupled respiration rates. However, very interestingly, by focusing on the first few hours of treatment, we observed that, the decrease in DNP-uncoupled respiration rate was significantly faster compared with the COX-dependent respiration rate decrease. In fact, after 1 hr of STS treatment, the TMPD-dependent respiration rate was decreased only by 4% relative to the rate of untreated cells, while the DNP-uncoupled respiration rate was decreased by 15% (Figure 1(b)). This difference remains constant with increasing time of STS-treatment. Since the COX-dependent respiration depends directly on cytochrome *c* and decreases less than the overall uncoupled respiration, this means that the uncoupled respiration is affected by some other modifications than the absence of cytochrome *c*. This finding suggests therefore that there is an impairment of the mitochondrial respiration soon after the apoptotic induction that is not due to the release of cytochrome *c*.

### 2.2. Temporal Correlation between Respiration Changes, Cytochrome *c* Release and Nuclear Fragmentation during STS-Induced Apoptosis; Uncoupled Respiration Drop Precedes Cytochrome *c* Release

To better understand the temporal correlation between decrease in respiration and cytochrome *c* release in STS-induced apoptosis we analyzed cytochrome *c* localization by confocal immunofluorescence microscopy. 2 uM STS-treatment of PC12 cells promoted nuclear fragmentation (Figure 2(a), STS 3 h; white arrow) and cytochrome *c* release from mitochondria as clearly resulted from the different immunolocalization of the mitochondrial marker Hsp60 and cytochrome *c* (Figure 2(a), STS 3 h; yellow arrows). Cell death was detected by DAPI staining and morphological analysis of nuclei. Figure 2(a) (white arrow) shows an example of cell considered apoptotic; it is shrunk and has a fragmented nucleus. By analyzing several fields, we calculated the percentage of cells showing cytochrome *c* release and the percentage of cells showing nuclear fragmentation (cell death) at each time point in order to compare these kinetics with the changes in respiration. Since cytochrome *c* release from mitochondria during apoptosis precedes nuclear fragmentation, we detected cells in which the cytochrome *c* had been released from mitochondria while the nucleus was not yet fragmented (Figure 2(a), STS 3 h; yellow arrowheads) but never the opposite. For this reason, we obtained a kinetics of cytochrome *c* release slightly more rapid than the kinetics of nuclear fragmentation (Figure 2(b)). Notably, once released from mitochondria, cytochrome *c* localizes throughout the cell and, as sometimes reported [16, 17], into the nucleus (Figure 2(a), STS 3 h; yellow arrows).

During the immunofluorescence staining, a high number of late stage apoptotic cells detached from the dish and were lost which means that we run the risk to underestimate their number. To solve this problem we calculated the real percentage of apoptotic cells by collecting all of them, also from the supernatant, through high speed centrifugation and by staining all nuclei with DAPI. The difference between the percentage of cell death calculated by immunofluorescence on a dish and the one obtained by high speed centrifugation was used to correct the underestimated percentage of cytochrome *c* releasing cells evaluated by means of the immunofluorescence staining; dead cells detaching during the experiment are at the very final stages of apoptosis and have already released cytochrome *c* [18].

We compared these kinetics with the changes in respiration reported in Figure 1, and for this purpose we expressed data shown in Figure 2(b) as percentage of cells with mitochondrially localized cytochrome *c* and percentage of alive cells (Figure 2(c)). We observed that the progressively lower rate of respiration upon STS treatment highly correlates with the decreased number of alive cells due to apoptotic death. Interestingly, the decrease of cells with mitochondrial cytochrome *c* localization highly correlates with the kinetics of COX-respiration decrease, but such a decrease is slower than that observed in DNP-respiration (Figure 2(c)). Such an analysis strengthens the conclusion that the decrease in TMPD-dependent respiration is mainly due to cytochrome *c* release, while the decrease in DNP-uncoupled respiration precedes cytochrome *c* release and must be due to another mechanism of impairment of the respiratory chain system.

### 2.3. Respiration Rates, Cytochrome *c* Localization, and Nuclear Changes in Bcl-2 Transfected Cells DNP-Dependent Respiration Drop Occurs Also in Absence of Cytochrome *c* Release

**Figure 3:**
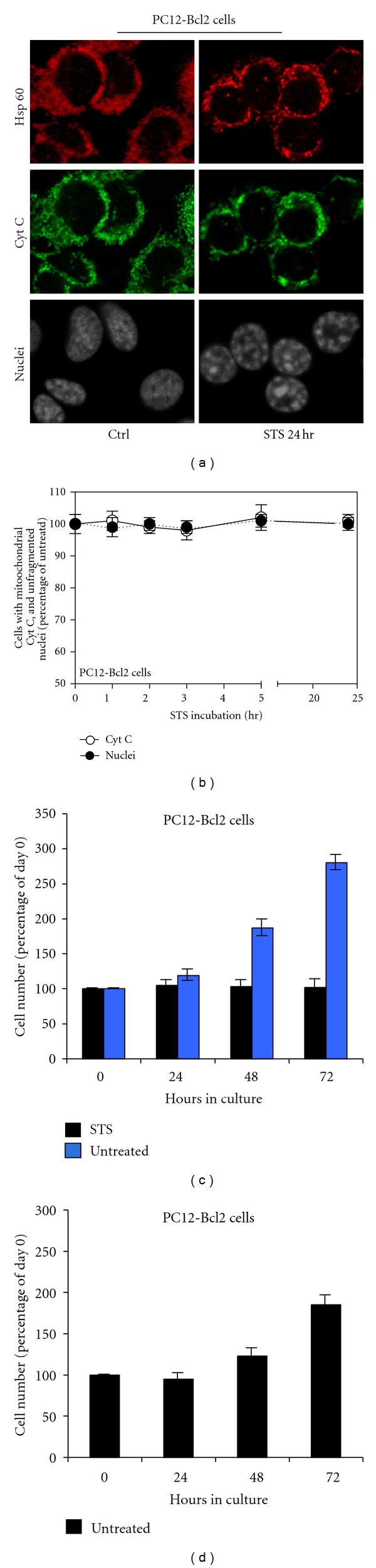
Cytochrome *c* localization and nuclear changes in STS-treated PC12-Bcl-2 cells. (a) Triple labeling confocal immunofluorescence microscopy of PC12-Bcl-2 cells untreated (Ctrl) and 2 *μ*M STS-treated for 24 h (STS 24 h). Pattern of Hsp60 (red), cytochrome *c* (green), and nuclear staining by SYTO-X (grey) of the same representative fields are shown. (b) Percentage of PC12-Bcl2 cells with mitochondrially localized cytochrome *c* and percentage of alive PC12-Bcl2 cells treated with STS for the indicated time. Data were calculated in 3 independent experiments for each time point. About 1000 cells were considered for each experiment at each time point. Note that the ordinate scale starts at 50%. (c) For quantification of cell proliferation, the same number of untreated or STS-treated PC12-Bcl2 cells was plated on several dishes and counted at the indicated times. Data were calculated in 3 independent experiments for each time point. (d) For quantification of cell proliferation, the same number of STS-treated PC12-Bcl2 cells were plated on several dishes and counted at the indicated times. At 24 h the medium was replaced in order to remove STS. Data were calculated in 3 independent experiments for each time point.

To confirm that the early changes in respiration observed during apoptosis were really independent of cytochrome *c* release, we decided to study the respiration of PC12 cells stably overexpressing the apoptotic inhibitor Bcl-2 (PC12-Bcl-2 cells), which is known to block the release of cytochrome *c* from mitochondria [19, 20]. In fact, PC12-Bcl-2 cells treated with 2 uM STS do not detach from the dish and do not undergo apoptosis as shown by DAPI staining (Figures 3(a) and 3(b), nuclei). Moreover, the release of cytochrome *c* from mitochondria is inhibited. Indeed, also after prolonged exposure to STS, cytochrome *c* and Hsp60 maintain the same localization in PC12-Bcl-2 cells (Figures 3(a) and 3(b)).

**Figure 4:**
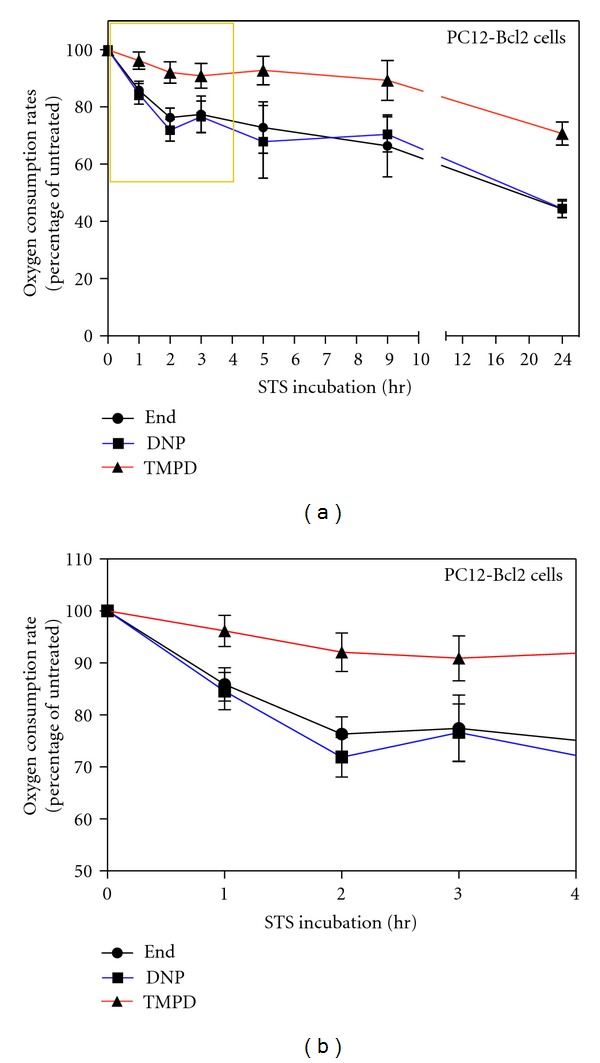
Decrease in endogenous, DNP-uncoupled, and TMPD-dependent respiration rates in intact PC12-Bcl-2 cells treated with STS; early decrease in DNP-uncoupled respiration. (a) Oxygen consumption rates of STS-treated PC12-Bcl-2 cells measured at the indicated time points. Oxygen consumption rates were measured in TD buffer (endogenous respiration, End), in TD buffer containing DNP (DNP-uncoupled respiration, DNP) and in TD buffer containing DNP, antimycin A, ascorbate and TMPD (TMPD-dependent respiration, TMPD). Data are expressed, at each time point, as percentages of the oxygen consumption of untreated cells (0 hr). The data represent the means ± SE with *n* varying at each time point and being 17, 20, 12, 2, 3, 6, 8 respectively for the indicated time points (1 h, 2 h, 3 h, 5 h, 9 h, 24 h). (b) magnification of (a) area surrounded by a yellow frame. Note that the ordinate scale starts at 50%.

The analysis of the respiration of these cells (Figure 4(a)) shows that Bcl-2 strongly protects against long-term respiratory decrease. In fact, after 24 h of treatment the uncoupled endogenous respiration decreases to ~44.5% of the rate of untreated cells while in absence of Bcl-2, under STS treatment, respiration decreased to ~16% of the rate of untreated cells (or even less considered the overestimation cited) (Figure 1(a)). The difference observed for the COX-dependent respiration is even greater; indeed, after 24 h of treatment, PC12-Bcl-2 cell respiration decreases only to ~70.7% of the untreated cell rate, while the COX-dependent oxygen consumption of naive PC12 cells falls to ~19.8% of the control. The protection of the respiratory activity by Bcl-2 is mainly due to the fact that Bcl-2 blocks the release of the cytochrome *c* from mitochondria (Figures 3(a) and 3(b)). However, the early decrease in DNP-dependent respiration that we have found during the first hours of apoptosis occurs also in Bcl-2-overexpressing cells, although cytochrome *c* is not released. Indeed, after 1 h of STS treatment, both in PC12-Bcl-2 and naive PC12 cells, DNP-uncoupled respiration decreases to ~84.6% of the respiration rate of untreated cells, and the COX-dependent respiration decreases only to ~96% of untreated cell respiration. After two hours of STS treatment, the uncoupled endogenous respiration falls to ~72% and ~74% of the control in PC12-Bcl-2 and naive PC12 cells, respectively, while the TMPD-dependent respiration reaches the 86% of the control in naive PC12 cells and remains almost unchanged in PC12-Bcl-2 cells (92%) (Figures 1 and 4).

Since in PC12-Bcl-2 cells cytochrome *c* is not released from mitochondria, this experiment confirms that, in the first phases of apoptosis, the maximum possible respiration rate achievable in the cell decreases and that the respiratory chain is impaired independently of the release of cytochrome *c*.

**Figure 5:**
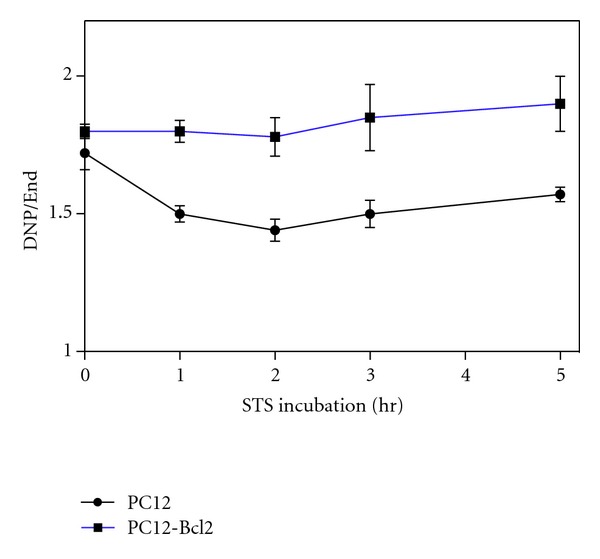
Uncoupling ratio behaviour in STS treated PC12 and PC12-Bcl-2 cells. Ratio between absolute DNP-uncoupled oxygen consumption and endogenous respiration expressed in nanomoles of oxygen consumed per minute and per mg of cellular proteins (nmolO_2_/min/mg) of PC12 and PC12-Bcl-2 cells under 2 *μ*M STS-treatment for the indicated time. In detail, for the indicated time points (0 h, 1 h, 2 h, 3 h, 5 h), endogenous respiration oxygen consumption values are 14,82; 13,99; 12,75; 10,12; 11,10 nmolO_2_/min/mg for PC12 cells and 15,82; 12,78; 10,94; 10,98; 9,81 nmolO_2_/min/mg for PC12-Bcl-2 cells. DNP-uncoupled oxygen consumption values are 25,50; 20,99; 18,36; 15,17; 17,43 nmolO_2_/min/mg and 28,48; 23,14; 19,48; 20,31; 18,64 nmolO_2_/min/mg.

Moreover, the comparison between the respiration of naive PC12 and PC12 cells overexpressing Bcl-2 also shows that the ratio between the absolute values of DNP-uncoupled and endogenous respiration (DNP/End) decreases in PC12 cells immediately after STS treatment, while, in cells overexpressing Bcl-2 remains unchanged (Figure 5). This suggests an early uncoupling respiration event induced by STS that is prevented by Bcl-2.

The DNP-dependent respiration is uncoupled from the synthesis of ATP since it occurs when there is a dissipation of the proton gradient which cannot be used to provide the energy for the synthesis of ATP. In this condition, the consumption of oxygen is the highest possible since it is free of control by the proton gradient and by the synthesis of ATP. DNP-dependent respiration rate is therefore higher than normal endogenous respiration since it is the maximum rate achievable by the respiratory chain. For this reason the ratio beween DNP-dependent respiration and endogenous respiration (DNP/End) is higher than 1. If the ratio between DNP and End decreases, this means that endogenous respiration of the cell tends to become uncoupled. In the case of PC12-Bcl2 cells the ratio remains constant which means that there is no uncoupling, while in PC12 cells this ratio decreases and this indicates an early uncoupling event (Figure 5).

### 2.4. Respiration Changes and Cytochrome *c* Release in STS-Treated PC12 Cells Are Not Dependent on Caspases and Do Rely Only on the Apoptotic Role of STS

**Figure 6:**
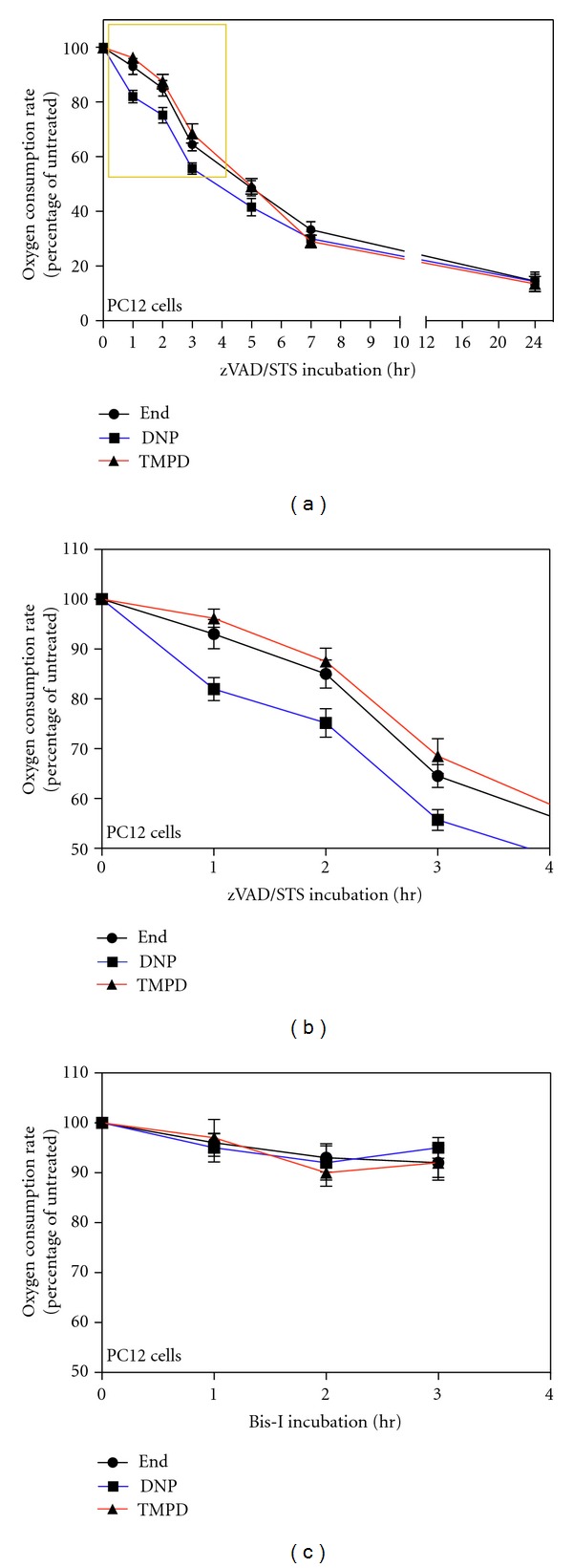
Decrease in endogenous, DNP-uncoupled, and TMPD-dependent respiration rates in STS-treated PC12 cells pretreated with z-VADfmk and in Bis-I-treated cells. (a) Relative oxygen consumption rates of 100 *μ*M zVADfmk-pretreated, 2 *μ*M STS-treated PC12 cells for the indicated times. Oxygen consumption rates were measured in TD buffer (endogenous respiration, End), in TD buffer containing DNP (DNP-uncoupled respiration, DNP) and in TD buffer containing DNP, antimycin A, ascorbate and TMPD (TMPD-dependent respiration, TMPD). Data are expressed, at each time point, as percentages of the oxygen consumption of untreated cells (0 hr). Data are expressed as means ± SE with *n* varying at each time point and being 8, 6, 6, 2, 3, 2, 3 for 1 h, 2 h, 3 h, 5 h, 9 h, and 24 h, respectively. (b) Magnification of the panel (a) area surrounded by a yellow frame. Note that the ordinate scale starts at 50%. (c) Relative oxygen consumption rates of Bis-I-treated PC12 cells for the indicated times. Data are expressed as means ± SE with *n* varying at each time point and being 5, 6, and 5 for 1 h, 2 h, and 3 h respectively.

In order to define whether the decrease of respiration, under STS treatment, was caspase dependent we pretreated PC12 cells with the broad-spectrum caspase inhibitor z-Val-Ala-Asp (OMe)-CH_2_F (z-VADfmk) before STS incubation. While z-VADfmk blocks apoptosis also after 24 h of STS treatment (Figure 2(a), zVAD-STS 6 h, nuclear morphology), it did not affect the extent of respiration rate reduction (Figures 6(a) and 6(b)). In fact, the decrease in endogenous, DNP-uncoupled and COX-dependent respiration upon STS treatment was perfectly comparable with the respiration rates measured in PC12 naive cells undergoing apoptosis (Figures 1(b)). Active caspases are indeed not required for the mitochondrial release of cytochrome *c* in STS-induced cell death [21]. In fact, the analysis of the localization of cytochrome *c* by confocal immunofluorescence over several hours after apoptotic induction shows a gradual release of cytochrome *c* not affected by zVADfmk pretreatment (Figure 2(a), zVAD-STS 6 h, arrows).

To exclude the possibility that the early reduction of respiratory activity we detected during the first phase of apoptosis was due to the mere protein kinase inhibition activity of STS, without any relationship with its apoptotic induction, we tested bisindolylmaleimide-I (Bis-I), an STS-analog which is a protein kinase inhibitor without apoptotic functions [22, 23]. The treatment of PC12 cells with Bis-I shows that this drug does not induce apoptosis (data not shown) and does not inhibit the respiration of cells (Figure 6(c)). The lack of respiratory changes in cells treated with Bis-I confirms that the decrease in respiration and the uncoupling of respiration induced by STS correlate with its apoptotic role.

### 2.5. Cytochrome *c* Independent and Reversible Nuclear Condensation Induced by STS

As stated before, STS-treated PC12-Bcl-2 do not die and do not show nuclear fragmentation typical of apoptotic cells also after long treatment (Figures 3(a) and 3(b)). However, despite the absence of fragmentation, we can clearly observe a strong condensation of chromatin (Figure 3(a)), which is very similar to the first stage of condensation observed in STS-treated PC12 cells and preceding fragmentation (Figure 2(a), STS 3 h, nuclei yellow arrowheads). STS-treated PC12-Bcl-2 cells bearing this strong nuclear condensation (Figure 3(a)) are alive since they respire (Figure 4) but do not replicate (Figure 3(c)); they do not die but stay in a quiescent state. In fact, when STS is removed by changing the medium, all nuclei regain a normal appearance and cells started replicating again (Figure 3(d)). We conclude that the first stage of nuclear modification in STS-induced apoptosis is not dependent on cytochrome *c* release and is not a point of no return of the apoptotic cascade.

## 3. Discussion

The overall decrease in respiration we detected during STS treatment of PC12 cells can obviously be related to the release of cytochrome *c*. However, we also found an impairment of the uncoupled respiration occurring in the first phase of STS-induced apoptosis and preceding the release of cytochrome *c*. This phenomenon was also reported in 143B cells [24], while in anti-Fas antibody-treated Jurkat cells, the loss of TMPD-dependent and of endogenous respiration occurred nearly simultaneously and followed cytochrome *c* release from mitochondria [15]. This difference in the sequence of events might rely on different pathways taking place in Fas- and STS-induced apoptosis.

Regarding the cause of this early respiration impairment independent of cytochrome *c* release, we hypothesize a modification of the mitochondrial inner membrane integrity likely to be present at the level of the phospholipid CL. Indeed, CL is intimately linked to the mitochondrial bioenergetic machinery and is also actively involved in the release of cytochrome *c*. It has also been demonstrated that the modifications of the binding between CL and cytochrome *c* and also the binding between CL and some Bcl-2 proteins precede and prime mitochondria for MOMP and for cytochrome *c* release [8]. CL is crucial for maintaining the integrity and the function of the mitochondrial inner membrane and is required for the optimal activity of most of the respiratory chain complexes. For these reasons, CL modifications (e.g., peroxidation by ROS and cytochrome *c* peroxidase activity or tBid binding or redistribution within and between membranes) immediately result in structural changes of the mitochondrial inner membrane which destabilize mitochondria and affect the activity of membrane embedded respiratory chain complexes [6]. This would impair respiration independently of cytochrome *c* release, as we found. Peroxidation of CL does occur following a wide variety of apoptotic stimuli also including STS. For all these reasons we hypothesize that the early changes in respirations observed might be due to perturbation of the mitochondrial membrane at the level of CL.

Our analysis also suggests that the first stage of apoptosis is characterized by an early uncoupling event. Indeed, in the first hours of STS treatment of PC12 cells, the ratio between the absolute DNP-uncoupled and the endogenous oxygen consumption decreases (Figure 5), indicating a difference between endogenous and DNP-uncoupled respiration decrease rate (Figure 1). This uncoupling event occurs after 1 h of STS treatment when TMPD respiration is only slightly affected, and it is therefore presumably independent of cytochrome *c* release. However, the uncoupling is blocked by the overexpression of Bcl-2 (Figures 4 and 5). Therefore, Bcl-2 does not prevent the early decrease in respiration induced by STS (Figure 4) but prevents the uncoupling of respiration, which might be the step following the decrease of the maximum rate of respiration and ultimately leading to cytochrome *c* release. We suggest that the uncoupling of respiration might correlate with the step of the cytochrome *c* release process involving the Bcl-2 family of proteins. Interestingly, the uncoupling of respiration is known to be also caused by CL modifications, supporting our hypothesis of CL as early target for apoptotic changes in mitochondria.

Our experiments, in addition, show a strong degree of nuclear condensation (never reaching fragmentation) occurring in absence of cytochrome *c* release (Figure 3(a)). This partial chromatin condensation might be the first step of nuclear modifications leading ultimately to fragmentation, and it has been reported to involve DNA double-strand breaks [25, 26]. We assessed that, in naïve PC12 cells, this early nuclear condensation occurring in Bcl2-overexpressing cells is reversible and that it is not a point of no return in the apoptotic pathway (Figures 3(c) and 3(d)). This confirms the finding that MOMP inhibitors allow cells with preapoptotic chromatin condensation to repair their DNA and to return to a normal nuclear morphology [26].

Finally, we observed that cytochrome *c* redistributed within the nuclei once lost its mitochondrial localization after induction of apoptosis, and, after long STS treatment, as previously reported [27], it became finally degraded (Figure 2(a), zVAD-STS 6 h, white arrow). No cells with cytochrome *c* localized only into the cytosol were detectable. This means that the nuclear localization of cytochrome *c* is quick, and indeed it occurs not only when the nuclei are fragmented but also when the chromatin is not yet fragmented or at a very early stage of condensation (Figure 2(a), yellow arrows). This nuclear cytochrome *c* accumulation was zVAD-independent (Figure 2(a), zVAD-STS 6 h).

In conclusion, we found that mitochondrial bioenergetics is perturbed previously and independently of the release of cytochrome *c* during apoptosis. This finding may shed new light on the mechanisms leading to the release of cytochrome *c*. Indeed, considering the interdependencies existing between bioenergetics and apoptosis [28], their coinvestigation in a holistic approach is strongly needed.

## 4. Material and Methods

### 4.1. Cell Line and Culture Conditions

 PC12 cells were cultured at 37°C in a 5% CO_2_ atmosphere in F-12 K (GIBCO) medium supplemented with 2 mM L-glutammine, 1,5 g/L sodium bicarbonate, 15% horse serum, 2.5% fetal bovine serum (FBS), and penicillin-streptomycin. For apoptotic induction, cells were seeded at 2×10^5^/mL into tissue culture dishes coated with 20 *μ*g/mL poly-L-lysine (Sigma-Aldrich). After 2 days the medium was replaced, and after 2 hours 2 *μ*M STS (Sigma-Aldrich) in DMSO was added. 100uM z-VADfmk (Kamiya Biochemicals) was added 30 min before STS. Bisindolylmaleimide GF 109203X was purchased from Calbiochem.

### 4.2. Quantification of Apoptosis

Apoptosis was assessed by nuclear morphology. At different time points upon STS treatment, both cell floating in the medium and cells adherent to the dish were collected by centrifugation, washed with PBS, fixed in 4% paraformaldehyde (PFA), and stained with 1 *μ*g/mL of the fluorescent double-strand DNA-binding dye 4,6-diamidino-2-2phenylindol (DAPI, Sigma-Aldrich) for 8 min. One drop of this preparation was analyzed on a slide by fluorescent microscopy. Cells with large nuclei containing uniformly stained chromatin were counted as alive cells, while cells containing fragmented nuclei and/or pyknotic nuclei were counted as dead cells. Values were obtained from 40–50 fields of 1-2 slides for a total of around 1000 cells/experiments. Each experiment was replicated 6-7 times with similar results.

### 4.3. Transfection

 A human Bcl-2*α* cDNA [19] was subcloned from SFFV-Bcl-2 n1 into the neomycin resistance marker-containing plasmid pcDEF3 [29] under the control of the EF-1a promoter and then used for transfection into PC12 cells by electroporation. Exponentially growing PC12 cells (10^7^) were resuspended in 0.8 mL of PBS, mixed with 18 mg of Bcl-2-pcDEF3 and 5 mg of pGL1, and incubated on ice for 10 min. Electroporation was performed with a BioRad gene pulser by using a setting of 960 mF and 300 V. Cells then were incubated on ice for 10 min and plated in F12-K medium supplemented with 15% horse serum and 2.5% FBS on collagen coated dishes (BIOCOAT). After 48 h stable transfectant overexpressing Bcl-2 (PC12-Bcl-2 cells) was selected by using 0.3 mg mL G418 (Geneticin; Invitrogen) for 2 weeks.

### 4.4. Measurement of Respiration Rates in Intact Cells

 PC12 cells were suspended in TD buffer (137 mM NaCl, 5 mM KCl, 0.7 mM Na_2_HPO_4_, 25 mM Tris-HCl pH 7.4 at 25°C) at 3 × 10^6^ cells/mL, and the respiration rate was measured in an oxygraph (Yellow Spring Instruments, model 5300). The same measurement was assessed after addition of 40 *μ*M DNP or DNP and 20 nM antimycin A (Sigma-Aldrich) or DNP, Antimycin A, 400 *μ*M TMPD (Fluka) and 10 mM ascorbate (Sigma-Aldrich) as described [12]. Oxygen consumption rate was expressed in nanomoles of oxygen consumed per minute and mg of cellular proteins (nmolO_2_/min/mg) as determined by the Bradford procedure (BioRad) and as percentage of the untreated PC12 cell respiration. The oxygen consumption rate measured in presence of cells, DNP, antimycin A, ascorbate, and TMPD was corrected by subtraction of the oxygen consumption rate due to the autooxidation of ascorbate/TMPD in absence of cells [15].

### 4.5. Confocal Immunofluorescence Microscopy

Cells were cultured on collagen-coated dishes at a concentration of 5 × 10^5^ cells/mL. After 2 days cells were treated with STS, then washed with PBS, fixed in 4% PFA in PBS for 10 min at RT, washed, permeabilized with PBS containing 0,4% Triton X-100 for 5 min, and washed again with PBS. After blocking the cells with PBS containing 2% horse serum (HS-PBS) for 30 min, they were incubated with mouse anticytochrome *c* monoclonal antibody 6H2.B4 (BD Pharmingen) diluted 1 : 300 in HS-PBS and rabbit anti-Hsp60 antiserum (StressGene) diluted 1 : 200 for 1 h at 37°C in a humidified chamber. Following 3 washes in HS-PBS (5 min each), the cells were incubated with FITC conjugated goat anti-mouse IgG (Jackson ImmunoResearch Laboratories) diluted 1 : 200 and lissamine-rhodamine conjugated goat anti-rabbit IgG (Jackson ImmunoResearch Laboratories) diluted 1 : 200 for 1 h at room temperature. The specimens were washed 3 min in HS-PBS and incubated with SYTOX Green (Molecular Probes) 25 nM for 15 min at room temperature. Cells were washed again 3 times (5 min each) in PBS, mounted in Fluoroguard antifade reagent (BioRad), and analyzed on a Zeiss 510 laser scanning microscope.

### 4.6. Immunoblot Analysis

Same amount of cell lysates were analyzed by 15% SDS-PAGE. Proteins were then transferred to a polyvinylidene difluoride (PVDF) membrane (BioRad), and the membrane was treated for 1 h with blocking solution (0.02% Tween 20, 0.02% NaN3, 5% nonfat milk in PBS) at room temperature. The membrane was then incubated for 4 h at 4°C with mouse anti-Bcl-2 monoclonal antibody (sc-7382) (Santa Cruz Biotechnologies) diluted 1 : 1000 in blocking solution. It was then washed 3 times in PBS (10 min each) and 1 time with washing solution (150 mM NaCl, 50 mM Tris-HCl pH 7.5) and then incubated for 1 h at RT with sheep anti-mouse IgG peroxidase-linked (Amersham) diluted 1 : 2000 in washing solution containing 5% nonfat dried milk. Finally the membrane was washed 5 times in washing solution (10 min each), and specific protein complexes were identified using Super Signal West PICO chemiluminescence reagent (Pierce Biotechnology) by autoradiography.

## References

[1] Liu X, Kim CN, Yang J, Jemmerson R, Wang X (1996). Induction of apoptotic program in cell-free extracts: requirement for dATP and cytochrome *c*. *Cell*.

[2] Li P, Nijhawan D, Budihardjo I (1997). Cytochrome *c* and dATP-dependent formation of Apaf-1/caspase-9 complex initiates an apoptotic protease cascade. *Cell*.

[3] Srinivasula SM, Ahmad M, Fernandes-Alnemri T, Alnemri ES (1998). Autoactivation of procaspase-9 by Apaf-1-mediated oligomerization. *Molecular Cell*.

[4] Cory S, Adams JM (2002). The BCL2 family: regulators of the cellular life-or-death switch. *Nature Reviews Cancer*.

[5] Llambi F, Moldoveanu T, Tait SWG (2011). A unified model of mammalian BCL-2 protein family interactions at the mitochondria. *Molecular Cell*.

[6] Gonzalvez F, Gottlieb E (2007). Cardiolipin: setting the beat of apoptosis. *Apoptosis*.

[7] Nomura K, Imai H, Koumura T, Kobayashi T, Nakagawa Y (2000). Mitochondrial phospholipid hydroperoxide glutathione peroxidase inhibits the release of cytochrome *c* from mitochondria by suppressing the peroxidation of cardiolipin in hypoglycaemia-induced apoptosis. *Biochemical Journal*.

[8] Kagan VE, Bayir HA, Belikova NA (2009). Cytochrome *c*/cardiolipin relations in mitochondria: a kiss of death. *Free Radical Biology and Medicine*.

[9] Gonzalvez F, Pariselli F, Jalmar O (2010). Mechanistic issues of the interaction of the hairpin-forming domain of tBid with mitochondrial cardiolipin. *PLoS One*.

[10] Liu J, Weiss A, Durrant D, Chi NW, Lee RM (2004). The cardiolipin-binding domain of Bid affects mitochondrial respiration and enhances cytochrome *c* release. *Apoptosis*.

[11] Leber B, Lin J, Andrews DW (2007). Embedded together: the life and death consequences of interaction of the Bcl-2 family with membranes. *Apoptosis*.

[12] Villani G, Attardi G (1997). In vivo control of respiration by cytochrome *c* oxidase in wild-type and mitochondrial DNA mutation-carrying human cells. *Proceedings of the National Academy of Sciences of the United States of America*.

[13] Jacobson MD, Burne JF, Raff MC (1994). Programmed cell death and Bcl-2 protection in the absence of a nucleus. *The EMBO Journal*.

[14] Castedo M, Hirsch T, Susin SA (1996). Sequential acquisition of mitochondrial and plasma membrane alterations during early lymphocyte apoptosis. *Journal of Immunology*.

[15] Hajek P, Villani G, Attardi G (2001). Rate-limiting step preceding cytochrome *c* release in cells primed for Fas-mediated apoptosis revealed by analysis of cellular mosaicism of respiratory changes. *The Journal of Biological Chemistry*.

[16] Luetjens CM, Kogel D, Reimertz C (2001). Multiple kinetics of mitochondrial cytochrome *c* release in drug-induced apoptosis. *Molecular Pharmacology*.

[17] Ruiz-Vela A, Gonzalez de Buitrago G, Martinez AC (2002). Nuclear Apaf-1 and cytochrome *c* redistribution following stress-induced apoptosis. *The FEBS Letters*.

[18] Munoz-Pinedo C, Guio-Carrion A, Goldstein JC, Fitzgerald P, Newmeyer DD, Green DR (2006). Different mitochondrial intermembrane space proteins are released during apoptosis in a manner that is coordinately initiated but can vary in duration. *Proceedings of the National Academy of Sciences of the United States of America*.

[19] Hockenbery D, Nunez G, Milliman C, Schreiber RD, Korsmeyer SJ (1990). Bcl-2 is an inner mitochondrial membrane protein that blocks programmed cell death. *Nature*.

[20] Yang J, Liu X, Bhalla K (1997). Prevention of apoptosis by Bcl-2: release of cytochrome *c* from mitochondria blocked. *Science*.

[21] Bossy-Wetzel E, Newmeyer DD, Green DR (1998). Mitochondrial cytochrome *c* release in apoptosis occurs upstream of DEVD-specific caspase activation and independently of mitochondrial transmembrane depolarization. *The EMBO Journal*.

[22] Toullec D, Pianetti P, Coste H (1991). The bisindolylmaleimide GF 109203X is a potent and selective inhibitor of protein kinase C. *The Journal of Biological Chemistry*.

[23] Harkin ST, Cohen GM, Gescher A (1998). Modulation of apoptosis in rat thymocytes by analogs of staurosporine: lack of direct association with inhibition of protein kinase C. *Molecular Pharmacology*.

[24] Duan S, Hajek P, Lin C, Shin SK, Attardi G, Chomyn A (2003). Mitochondrial outer membrane permeability change and hypersensitivity to digitonin early in staurosporine-induced apoptosis. *The Journal of Biological Chemistry*.

[25] Leist M, Jaattela M (2001). Four deaths and a funeral: from caspases to alternative mechanisms. *Nature Reviews Molecular Cell Biology*.

[26] Andreau K, Castedo M, Perfettini JL (2004). Preapoptotic chromatin condensation upstream of the mitochondrial checkpoint. *The Journal of Biological Chemistry*.

[27] Ferraro E, Pulicati A, Cencioni MT (2008). Apoptosome-deficient cells lose cytochrome *c* through proteasomal degradation but survive by autophagy-dependent glycolysis. *Molecular Biology of the Cell*.

[28] Ricci JE, Gottlieb RA, Green DR (2003). Caspase-mediated loss of mitochondrial function and generation of reactive oxygen species during apoptosis. *Journal of Cell Biology*.

[29] Goldman LA, Cutrone EC, Kotenko SV, Krause CD, Langer JA (1996). Modifications of vectors pEF-BOS, pcDNA1 and pcDNA3 result in improved convenience and expression. *Biotechniques*.

